# Annotations

**Published:** 1895-06-15

**Authors:** 


### ANNOTATIONS.

### Heart Troubles at Halifax.

Medical officers of health are makers, not only of "history," but of "working science." For example, Dr. Ainley, M.O.H. for Halifax, tells us that heart affections are "a constantly increasing factor" in the mortality returns, as indeed is well recognised. These are his words: "Whatever the cause of the increased number of deaths from heart disease, it is very obviously a constantly increasing factor; the lives we are saving in the zymotic class we are losing in those who die from heart troubles, and probably also from increasing nervous diseases; no doubt there is truth in many of the theories advanced, such as the hurry and worry of modern life." Now, these are very significant words indeed; for, if we were asked to choose between death by quickly operating epidemics, and death by the slow process of heart impairment, or the equally slow and distressing breakdown of the nervous system, we, at any rate, would unhesitatingly choose the quick and comparatively painless epidemic. Nothing worse can well be imagined from the point of view of the general well-being and happiness than a large increase in the number of heart-disease cases, and of those illnesses which are directly due to "hurry and worry." We hope that all medical officers of health and others whose business it is to compile vital statistics, will direct their special attention to this class of facts for a few years, in order that special measures shall be taken by the public medical and sanitary authorities to bring about conditions of life more conducive to general happiness and well-being.

### The General Medical Council and the Midwives Bill.

The General Medical Council has discussed the Midwives Bill. The alterations suggested are not, however, of a very important character, and in no way alter the general tenor of the Bill, which remains as it was—a Bill to prevent anyone assuming the title of midwife except under certain conditions, and to provide a midwives board, who shall determine these conditions and publish a register of those women who conform with them. It is a curious instance of the general ineptitude of the Medical Council that although this Bill had been referred to them by the Lord President of the Privy Council for their opinion, it never seems to have occurred to them to urge the one great and fatal objection to it, viz., that it will be inoperative for the object it has in view. This object was well described by Dr. Glover, who said it was "the protection of women from the incompetent," and if the amendments and modifications proposed in the Council are considered *seriatim*, it soon becomes clear how little this fundamental basis, on which alone legislation is asked for, entered into the discussions of that body. The Bill puts no obstacle in the way of the practice of midwifery by any who choose to practise it, only they must not call themselves midwives. What, then, are we to think of amendments which aimed at substituting the term "midwifery nurses" for "midwives"? The Bill is weak enough as it is in regard to any prevention of unqualified practice, but the proposal made by the committee of the Council to make it protective only of the new and unknown term, "midwifery nurse," a term which is not coveted by anyone, and which no one would adopt, while all the time not only the practice of midwifery, but even the assumption of the name of midwife, would be left open to everyone, was so obviously absurd as to lead to its rejection. It is worth noting, however, that no suggestion was made to the Lord President that an extension of the restrictions of the Bill to cover the *practice* of midwifery for gain was necessary if its object was to be attained, viz., "the protection of women from the incompetent." Few things could be more ridiculous than to frame a Bill for the prevention of unqualified practice upon the lines of the Medical Acts, which are notoriously incompetent for that purpose, unless it be the proposal of Sir Walter Foster to create a title, "midwifery nurse," which nobody asks to have, and then to devote all the energies of an Act of Parliament to its protection. In dealing with this question, it should never be forgotten that all Acts of Parliament regulating the assumption of titles are absolute failures in regard to regulating practice. Unqualified medical practice is in no way interfered with by the Medical Acts, and yet it is proposed again to enact the same farce, and, on the sole plea of protecting women from the dangers arising from their being attended by unqualified midwives, to pass an Act dealing merely with the assumption of a particular title. If it is desired to put a stop to the practice of midwifery by ignorant, dirty, and incompetent women, the only way is to prohibit it. A simple Act rendering it a misdemeanour for anyone, after a certain date, to practise midwifery for gain, unless possessed of a certificate of competence; providing a very small Midwives Board to decide from time to time by whom such certificates should be granted; and putting the whole matter of the suppression of unqualified practice into the hands of the police—would at once give to the women of England the protection which is required. All this talk about titles is mere child's play.

### Anti-toxin for Snake-bite.

The results of his investigations regarding the treatment of snake-bite, which Professor Frazer has just communicated to the Royal Society of Edinburgh, are full of interest, especially at the present time, following as they do, in other fields, the same line that has been taken in supplying anti-toxin for the treatment of zymotic diseases, such as diphtheria. Shortly, the process he has adopted has been the establishment of "toleration" in an animal by the administration of gradually-increasing doses of snake poison. Beginning with very small, non-fatal doses, he found that the animals experimented on showed a progressively-increasing immunity; and when that had been developed to a high degree it was found that, as in the case of diphtheria, the serum of these animals not only inhibited the action of the venom in others, but that, even when animals had received an otherwise fatal dose, the action of the poison could be stopped by injecting this anti-toxic serum, or, as Professor Frazer calls it, "antivenine." If this antivenine can be procured in quantity, it seems likely to prove of immense importance in India, where 20,000 persons annually die of snake-bite.

